# Reactivation of mutant p53 by a dietary-related compound phenethyl isothiocyanate inhibits tumor growth

**DOI:** 10.1038/cdd.2016.48

**Published:** 2016-06-03

**Authors:** M Aggarwal, R Saxena, E Sinclair, Y Fu, A Jacobs, M Dyba, X Wang, I Cruz, D Berry, B Kallakury, S C Mueller, S D Agostino, G Blandino, M L Avantaggiati, F-L Chung

**Affiliations:** 1 Department of Oncology, Lombardi Comprehensive Cancer Center, Georgetown University, Washington, DC 20007, USA; 2 Department of Biochemistry and Molecular and Cellular Biology, Georgetown University, Washington, DC 20007, USA; 3 National Institutes of Arthritis and Musculoskeletal and Skin Diseases, National Institutes of Health, Bethesda, MD 20892, USA; 4 Translational Oncogenomics Unit, Italian National Cancer Institute 'Regina Elena', Rome, Italy

## Abstract

Mutations in the p53 tumor-suppressor gene are prevalent in human cancers. The majority of p53 mutations are missense, which can be classified into contact mutations (that directly disrupts the DNA-binding activity of p53) and structural mutations (that disrupts the conformation of p53). Both of the mutations can disable the normal wild-type (WT) p53 activities. Nevertheless, it has been amply documented that small molecules can rescue activity from mutant p53 by restoring WT tumor-suppressive functions. These compounds hold promise for cancer therapy and have now entered clinical trials. In this study, we show that cruciferous-vegetable-derived phenethyl isothiocyanate (PEITC) can reactivate p53 mutant under *in vitro* and *in vivo* conditions, revealing a new mechanism of action for a dietary-related compound. PEITC exhibits growth-inhibitory activity in cells expressing p53 mutants with preferential activity toward p53^R175^, one of the most frequent ‘hotspot' mutations within the p53 sequence. Mechanistic studies revealed that PEITC induces apoptosis in a p53^R175^ mutant-dependent manner by restoring p53 WT conformation and transactivation functions. Accordingly, in PEITC-treated cells the reactivated p53^R175^ mutant induces apoptosis by activating canonical WT p53 targets, inducing a delay in S and G2/M phase, and by phosphorylating ATM/CHK2. Interestingly, the growth-inhibitory effects of PEITC depend on the redox state of the cell. Further, PEITC treatments render the p53^R175^ mutant sensitive to degradation by the proteasome and autophagy in a concentration-dependent manner. PEITC-induced reactivation of p53^R175^ and its subsequent sensitivity to the degradation pathways likely contribute to its anticancer activities. We further show that dietary supplementation of PEITC is able to reactivate WT activity *in vivo* as well, inhibiting tumor growth in xenograft mouse model. These findings provide the first example of mutant p53 reactivation by a dietary compound and have important implications for cancer prevention and therapy.

Mutations in the p53 gene occur in a variety of human cancers with remarkably high frequencies (www-p53.iarc.fr). The majority of p53 mutations are missense that are localized to six ‘hotspot' residues. Mutations in p53 result in the loss of the wild-type (WT) activity; however, these mutants exert either a ‘dominant-negative' effect on the p53 WT activity or a ‘gain-of-function' effects.^1, 2, 3^ Humans with a Li–Fraumeni syndrome, an autosomal-dominant disorder owing to germline mutations in p53 gene, are at an increased risk of tumorigenesis.^4^ Thus targeting p53 mutant offers a promising approach for cancer chemotherapeutics. However, the role of p53 mutant as a target for dietary-related cancer chemopreventive compounds remained to be investigated.

Phenethyl isothiocyanate (PEITC), abundantly present in watercress and cruciferous vegetables, exerts cancer chemopreventive effects in animal models, and epidemiological studies also support the role of dietary ITCs in protection against cancer in humans.^5^ In fact, PEITC has been studied in clinical phase 1 and phase 2 trials (http://www.clinicaltrials.gov/ct2/results?term=PEITC). The mechanisms proposed for PEITC include inhibition of cytochrome P450s, induction of phase II detoxifying enzymes, cell cycle arrest and apoptosis.^6, 7, 8, 9, 10, 11, 12^ PEITC-induced oxidative stress contributes to apoptosis;^13, 14^ however, the exact mechanism(s) underlying its activity and its molecular target(s) are not well understood. This knowledge is crucial for discovering more effective ITCs for the prevention and treatment of cancer. In this study, we investigated p53 mutant as a new target of PEITC-induced apoptosis and tumor suppression.

## Results

### Effects of PEITC on proliferation of cells expressing p53 mutant

**Figure 1:**
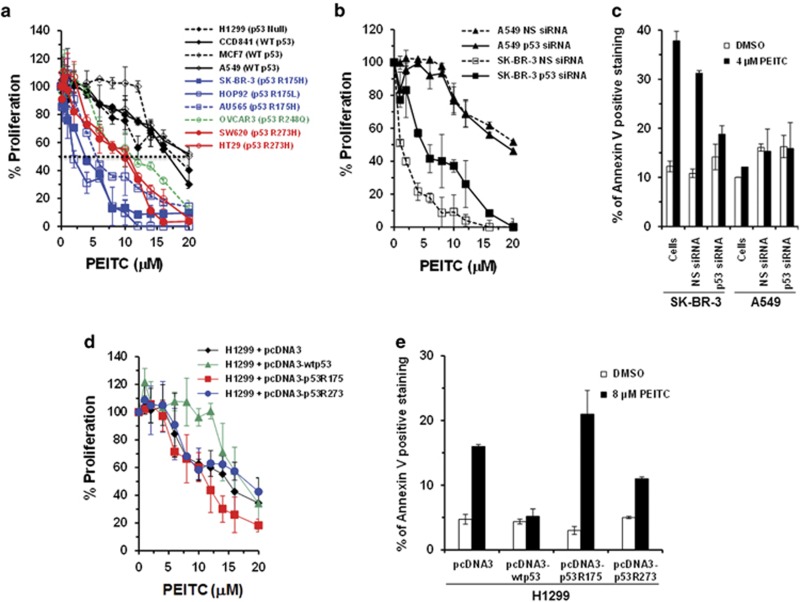
PEITC inhibits cell proliferation and induces apoptosis in a p53^R175^ mutant-dependent manner. (**a**) Human tumor cells lines with hotspot p53 mutations and p53 WT were treated with DMSO (control) or PEITC for 3 days. (**b**) SK-BR-3 and A549 cells transfected with siRNA were treated with DMSO or PEITC for 3 days. Percentage of cell proliferation was determined by the WST-1 assay. (**c**) Effect of PEITC on apoptosis. Untransfected (cells) or siRNA-transfected SK-BR-3 and A549 cells were treated with DMSO or 4 *μ*M PEITC for 3 days. Apoptosis was measured by Annexin-V staining using a BD LSRFORTESSA instrument. (**d**) The H1299 cells transfected with pcDNA3, pcDNA3-p53R175, pcDNA3-p53R273 or pcDNA3-wtp53 were treated with DMSO or PEITC for 3 days. Percentage of cell proliferation was determined by the WST-1assay. (**e**) Effect of PEITC on apoptosis. The H1299 cells transfected with pcDNA3, pcDNA3-p53R175, pcDNA3-p53R273 or pcDNA3-wtp53 were treated with DMSO or 8 *μ*M PEITC for 3 days. Apoptosis was measured by Annexin-V staining using a BD LSRFORTESSA instrument

We examined the effects of PEITC in tumor cells harboring mutations at the hotspot codons 175, 248 and 273. PEITC reduced proliferation of cells expressing different p53 mutants; however, maximal inhibition was observed in SK-BR-3, HOP92 and AU565 cells, which all express the p53^R175^ mutant (Figure 1a). In these cancer cells, PEITC exhibited IC_50_s that were ∼2.5–5-fold lower than in cells with other hotspot mutations. No significant inhibition of proliferation was observed in cells harboring a p53 WT treated with PEITC.

### PEITC inhibits proliferation and induces apoptosis in a p53^R175^-dependent manner

To determine whether the antiproliferative effects of PEITC are mediated through the reactivation of p53^R175^, we used cells transfected with control (NS) siRNA or p53 siRNA. The p53 protein was reduced by ≥90% after p53 siRNA transfection (Supplementary Figure S1a). In SK-BR-3 (Figure 1b), HOP92 (Supplementary Figure S1b) or AU565 (Supplementary Figure S1c) cells, the p53^R175^ knockdown resulted in markedly reduced sensitivity to growth inhibition by PEITC, whereas cells transfected with NS siRNA remained highly sensitive. No significant difference in proliferation was observed in A549 cells transfected with p53 siRNA or NS siRNA (Figure 1b). These results demonstrate that PEITC-induced growth inhibition is, at least partially, dependent on p53^R175^.

SK-BR-3 cells treated with 4 *μ*M PEITC displayed an approximately threefold increase in the percentage of Annexin-V-stained cells (Figure 1c), compared with MDA-MB-231(p53^R280^), OVCAR3 (p53^R248^) (Supplementary Figure S1d) or A549 cells (Figure 1c). Importantly, there was no significant difference in apoptosis in p53^R175^ knockdown SK-BR-3 cells treated with PEITC or DMSO (Figure 1c), demonstrating that PEITC-induced apoptosis is p53^R175^ dependent.

To validate our results, we treated isogenic human H1299 (p53 null) cells transfected with plasmid pcDNA3, pcDNA3-p53R175, pcDNA3-p53R273 or pcDNA3-wtp53 with PEITC. The H1299-pcDNA3-p53R175 cells displayed maximum sensitivity to PEITC (Figures 1d and e). We used mouse embryonic fibroblast (MEF) cells lacking p53 gene (10)3, (10)3 cell-line-derived mutant p53 transfectants ((10)3/175 and (10)3/273) and WT p53 Balb/c 3T3 cells. Consistent with previous results, the p53^R175^ mutant was the most sensitive to PEITC (Supplementary Figures S1e and f). Collectively these results provide strong support to the notion that PEITC-induced growth inhibition preferentially occurs in cells harboring p53^R175^.

### PEITC restores a ‘WT-like' conformation and transactivation functions to p53^R175^

**Figure 2:**
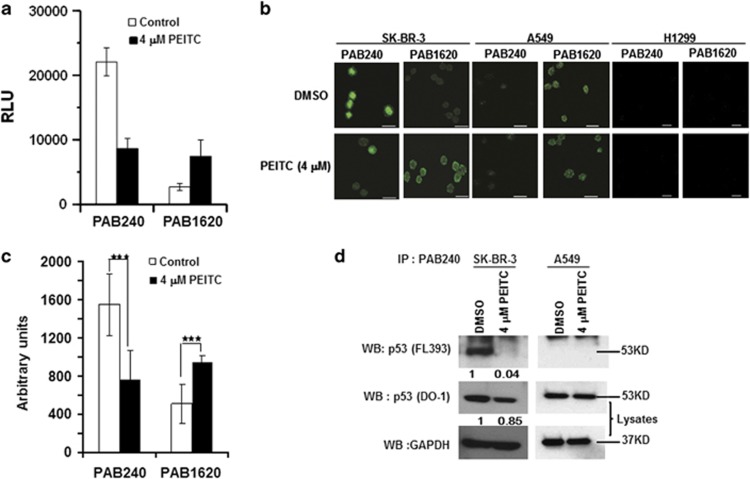
PEITC induces a ‘WT-like' conformational change in p53^R175^ mutant protein. (**a**) ELISA to determine the effect of PEITC on conformation of recombinant-purified GST-p53^R175H^ by using conformation-specific antibodies PAB240 (mutant-specific) and PAB1620 (WT-specific). (**b**) SK-BR-3 cells were treated with DMSO or 4 *μ*M PEITC for 6 h. Immunofluorescence of the cells was performed using PAB240 and PAB1620 antibodies. The A549 cell line used as a control showed that p53 WT conformation was not changed by PEITC. The H1299 cell line was used as a control for anti-p53 antibodies. All scale bars represents a size of 20 *μ*m. (**c**) Quantification of PAB240 and PAB1620 staining shown in panel (**b**). ***P≤0.0001 for PAB240 and PAB1620. (**d**) Immunoprecipitation of the p53 mutant protein from SK-BR-3 cell lysates using PAB240 antibody and detected by p53 (FL393) antibody

As PEITC induced apoptosis in a p53 mutant-dependent manner, we reasoned that it may do so by restoring p53 WT function(s). Therefore, we examined its effect on the conformation of p53^R175^ with enzyme-linked immunosorbent assay (ELISA) using conformation-specific anti-p53 antibodies. Incubation of the recombinant glutathione-*S*-transferase (GST)–p53^R175H^ with PEITC resulted in a ∼2.8-fold increase in the PAB1620 (WT-specific) fraction, whereas the PAB240 (mutant-specific) fraction was decreased by ∼2.6-fold (Figure 2a). An immunofluorescence assay on PEITC-treated SK-BR-3 cells showed an approximately twofold increase in the fluorescent intensity of PAB1620 antibody, whereas PAB240 antibody reactivity decreased (Figures 2b and c). Similar results were obtained with 10(3)/175 cells (Supplementary Figure S2a). Importantly, immunoprecipitation of p53^R175^ from SK-BR-3 (Figure 2d) and HOP92 (Supplementary Figure S2b) cells treated with PEITC revealed a >95% decrease in the PAB240 immunoreactivity. These results demonstrate that PEITC induced a ‘WT-like' conformation in p53^R175^.

**Figure 3:**
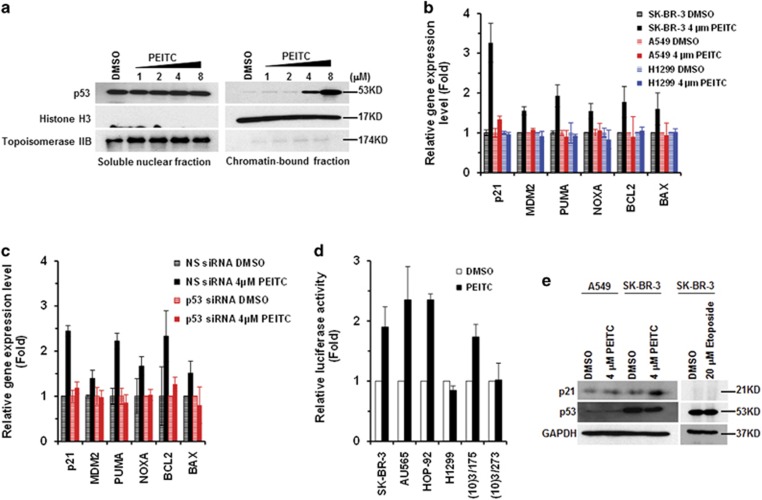
PEITC restores the p53^R175^ mutant protein transactivational functions. (**a**) PEITC induced p53^R175^ mutant protein to bind chromatin. SK-BR-3 cells were treated with PEITC for 4 h and chromatin-bound and nuclear-soluble fractions were analyzed by immunoblotting. Histone H3 and Topoisomerase IIB served as markers for the chromatin and soluble nuclear fractions, respectively. (**b**) qRT-PCR of p53 regulated genes in SK-BR-3, H1299 and A549 cells treated with DMSO or 4 *μ*M PEITC for 4 h. RNA was extracted and gene expression level was measured using TaqMan gene expression assay. (**c**) qRT-PCR of p53-regulated genes in NS siRNA or p53 siRNA-transfected SK-BR-3 cells treated with DMSO or 4 *μ*M PEITC for 4 h. RNA was extracted and gene expression level was measured using TaqMan gene expression assay. (**d**) SK-BR-3, HOP92, AU565, H1299 and MEF ((10)3/175 and (10)3/273) cells were transfected with plasmid 16451 and were treated with PEITC (4 or 6 *μ*M) for 24 h, respectively, followed by a luciferase reporter assay. (**e**) Western blotting analysis of p21 expression in SK-BR-3 cells treated with 4 *μ*M PEITC or 20 *μ*M etoposide for 4 h. A549 cell line was treated with 4 *μ*M PEITC for 4 h as a control. Protein levels were determined by western blotting using p21, p53 DO-1 and GAPDH antibodies

DNA binding is critical for p53 functions. We examined whether PEITC enriches p53^R175^ in the chromatin fractions. The chromatin-bound fractions of SK-BR-3 cells treated with PEITC showed a dose-dependent increase in p53^R175^ (Figure 3a). Consistent with this, PEITC (4 *μ*M) enhanced the expression of canonical p53 target genes, specifically p21, MDM2, PUMA, NOXA, BCL2 and BAX in SK-BR-3 cells (Figure 3b). No significant change was observed in A549, H1299 or p53^R175^ knockdown SK-BR-3 cells treated with PEITC (4 *μ*M), suggesting that the induction of p53 targets by PEITC was p53^R175^ dependent (Figures 3b and c). Finally, we performed luciferase reporter assay. PEITC (4 *μ*M)-treated SK-BR-3, AU565, HOP92 and (10)3/175 cells, transfected with plasmid encoding WT p53-binding element in the p21 promoter region, showed an ∼2–2.5-fold increase in luciferase activity (Figure 3d). PEITC (4 *μ*M) induced p21 expression in the SK-BR-3 cells, whereas the DNA-damaging agent etoposide failed to do so (Figure 3e), suggesting that the induction was p53^R175^ dependent. These results demonstrate that PEITC restores a ‘WT-like' conformation and transactivation function to p53^R175^.

### p53^R175^ protein undergoes degradation by the proteasome and autophagy

**Figure 4:**
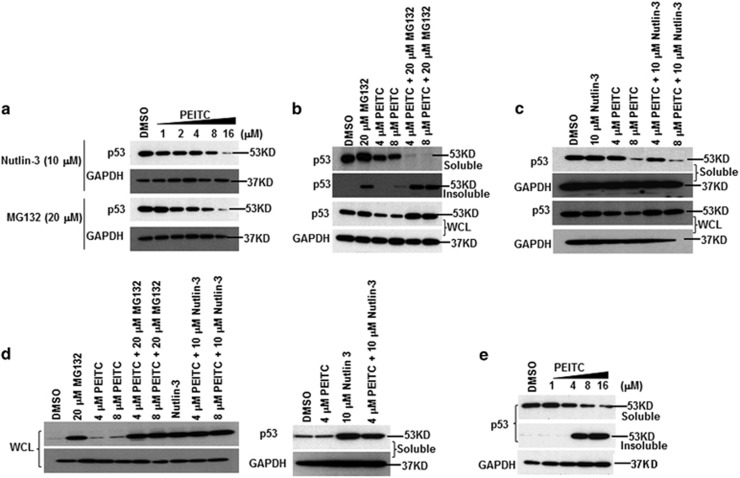
Proteasome degradation of p53 protein upon PEITC treatment in SK-BR-3 and A549 cells. (**a**) SK-BR-3 cells were treated with the indicated concentrations of PEITC and inhibitor (10 *μ*M Nutlin-3 or 20 *μ*M MG132) for 4 h. (**b**) SK-BR-3 cells were treated with PEITC (4 or 8 *μ*M), 20 *μ*M MG132 or both for 4 h. (**c**) SK-BR-3 cells were treated with PEITC (4 or 8 *μ*M), 10 *μ*M Nutlin-3 or both for 4 h. (**d**) A549 cells were treated with PEITC (4 or 8 *μ*M), inhibitor (10 *μ*M Nutlin-3 or 20 *μ*M MG132) or both for 4 h. Cells were harvested and lysates were prepared. Lysate fractions were resolved by SDS-PAGE and probed with p53 DO-1 antibody. (**e**) SK-BR-3 cells were treated with the indicated concentrations of PEITC or DMSO for 4 h. Cells were harvested and soluble and insoluble fractions were prepared. Thirty μg of the soluble and insoluble lysate fractions were resolved by SDS-PAGE and probed with p53 DO-1 antibody

PEITC (≥10 *μ*M) selectively depletes p53 mutant protein, but not the WT p53, although the mechanisms of such depletion are still unknown.^15^ Previous studies have shown that compounds that reactivate mutant p53 also induce its partial depletion.^16^ To investigate the molecular mechanisms for p53^R175^ depletion, we evaluated p53 mutant levels with different doses and times of PEITC treatment. PEITC induced significant decrease of p53^R175^ levels, at the concentration as low as 4 *μ*M, (Supplementary Figures S3a–c). As PEITC restored p53^R175^ to the ‘WT-like' form and p53 WT is regulated by the MDM2, the decreased stability of the restored p53^R175^ might be due to the MDM2-dependent proteasome degradation.^17^ To test this, SK-BR-3 cells were co-treated with the proteasome inhibitor MG132 or Nutlin-3, a specific MDM2 inhibitor, and PEITC. MG132 or Nutlin-3 were unable to prevent the decrease in p53^R175^ (Figure 4a). Studies have shown that inhibition of proteasome-dependent degradation of mutant p53 led to the accumulation of the ubiquitinated protein in the insoluble fraction.^18^ SK-BR-3 cells co-treated with PEITC (4 or 8 *μ*M) and 20 *μ*M MG132 showed a significant accumulation of the p53 in the insoluble fractions as well as in the whole-cell lysate (WCL) as compared with cells treated with PEITC or MG132 alone (Figure 4b). Similarly, SK-BR-3 cells co-treated with PEITC (4 or 8 *μ*M) and 10 *μ*M Nutlin-3 displayed an increase in p53 in the WCL (Figure 4c), whereas no difference in p53 accumulation was observed in A549 (Figure 4d). These results demonstrate that the reduced stability of the reactivated p53^R175^ is due to the proteasomal degradation.

**Figure 5:**
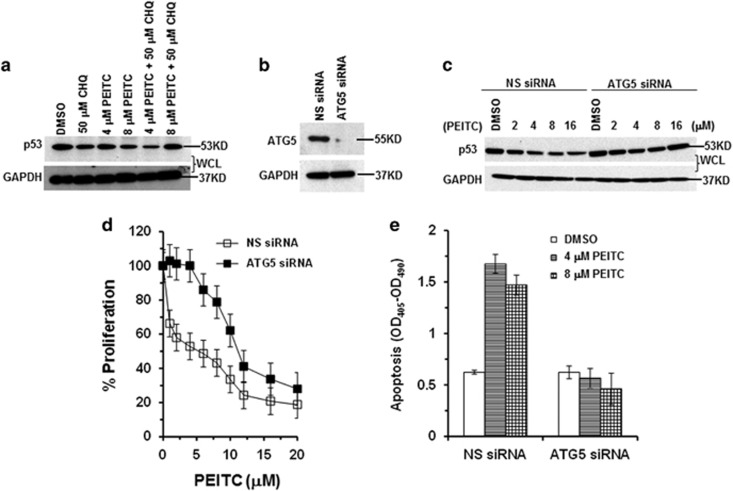
Autophagy of p53^R175^ protein upon PEITC treatment in SK-BR-3 cells. (**a**) SK-BR-3 cells were treated with PEITC (4 or 8 *μ*M), CHQ (50 *μ*M) or both for 4 h. Cell lysate fractions were resolved by SDS-PAGE and probed with p53 DO-1 antibody. (**b**) SK-BR-3 cells were transfected with ATG5 siRNA or NS siRNA. Thirty μg of the cell lysate was resolved by SDS-PAGE and probed with anti-ATG5 antibody. Blots were stripped and reprobed with anti-GAPDH antibody. (**c**) SK-BR-3 cells transfected with ATG5 siRNA or NS siRNA were treated with DMSO or PEITC for 4 h. Protein levels were determined by western blotting using p53 DO-1 and GAPDH antibodies. (**d**) SK-BR-3 transfected with ATG5 siRNA or NS siRNA were treated with DMSO or PEITC for 3 days. Percentage of cell proliferation was determined by the WST-1 assay. (**e**) Effect of PEITC on apoptosis. ATG5 siRNA- or NS siRNA-transfected SK-BR-3 cells were treated with DMSO or PEITC for 3 days. Cells were assayed for histone-associated DNA fragments indicative of apoptosis

SK-BR-3 cells treated with 8 *μ*M PEITC alone showed p53 in the insoluble fraction (Figure 4b). The insoluble fractions are known to contain protein aggregates.^19^ To gain insights on aggregation of the p53^R175^, we treated SK-BR-3 cells with broad range (1–16 *μ*M) of PEITC. Aggregation of p53^R175^ occurs at higher concentrations (≥8 *μ*M) (Figure 4e). As protein aggregates are cleared by autophagy, we investigated whether p53^R175^ undergoes autophagy. SK-BR-3 cells co-treated with 8 *μ*M PEITC and 50 *μ*M chloroquine (CHQ), an inhibitor of autophagy, displayed an increase of p53 in the WCL compared with the cells treated with PEITC alone. No significant difference was observed for cells co-treated with 4 *μ*M PEITC and 50 *μ*M CHQ or PEITC alone, suggesting that autophagy was induced at higher concentrations of PEITC (Figure 5a). Autophagy protein 5 (ATG5) is required for autophagosome formation.^20^ ATG5-knockdown SK-BR-3 cells (Figure 5b) showed higher levels of p53^R175^ at 8 or 16 *μ*M but not at 2 or 4 *μ*M concentrations of PEITC compared with cells transfected with NS siRNA (Figure 5c). ATG5-knockdown SK-BR-3 cells were resistant to the antiproliferative effects of PEITC, whereas NS siRNA-transfected cells remained highly sensitive (Figures 5d and e), suggesting that in these cells autophagy negatively regulates cell growth. Together, these results demonstrate that PEITC depletes the p53 mutant from the cells by two different pathways: MDM2-dependent proteasome degradation for the reactivated p53 mutant and autophagy for the p53^R175^ aggregates.

### Effects of zinc and redox changes on PEITC-induced p53^R175^ reactivation

**Figure 6:**
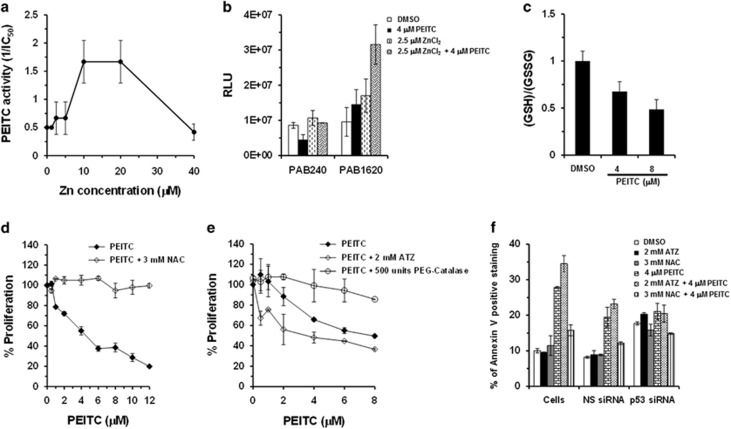
Effects of zinc and redox changes on PEITC-induced p53^R175^ reactivation. (**a**) Effect of zinc on the activity of PEITC. SK-BR-3 cells were treated with PEITC, zinc or both. Percentage of cell proliferation was determined by the WST-1 assay. The PEITC activity is shown as 1/IC_50_ for growth inhibition. (**b**) ELISA to determine the effect of zinc alone or zinc and PEITC on conformation of recombinant-purified GST-p53^R175H^ by using conformation-specific antibodies PAB240 (mutant-specific) and PAB1620 (WT-specific). (**c**) Effect of PEITC on the levels of reduced glutathione in SK-BR-3 cells. SK-BR-3 cells were treated with PEITC (4 or 8 *μ*M) or DMSO for 4 h. Ratio of reductant GSH and oxidative GSSG was then measured using the GSH/GSSG-Glo Glutathione Assay Kit. (**d**) Effect of NAC on PEITC activity. SK-BR-3 cells were treated with the indicated concentrations of PEITC or PEITC in combination with 3 mM NAC for 3 days. Percentage of cell proliferation was determined by the WST-1 assay. (**e**) SK-BR-3 cells were co-treated with the PEITC alone or in combination with 2 mM ATZ or 500 units PEG-Catalase for 3 days. Percentage of cell proliferation was determined by the WST-1 assay. (**f**) Effect on apoptosis. Untransfected (cells) or siRNA-transfected SK-BR-3 cells were treated with DMSO, ATZ, NAC or PEITC alone or PEITC in combination with ATZ or NAC for 3 days. Apoptosis was measured by Annexin-V staining using a BD LSRFORTESSA instrument

Zinc ion is required for the proper folding of p53 WT protein. p53^R175^ is incapable of binding to zinc.^21^ As PEITC restored the ‘WT-like' conformation to p53^R175^, we assessed the effect of zinc on its antiproliferative activity. Co-treatment of SK-BR-3 cells with PEITC and zinc chloride (ZnCl_2_) in the optimal concentration range (10–20 *μ*M) enhanced the potency of PEITC by ∼3.3-fold (Figure 6a) but not with ZnCl_2_ alone. Also, incubation of the GST-p53^R175H^ with PEITC (4 *μ*M) and ZnCl_2_ (2.5 *μ*M) resulted in significant increase in the PAB1620 fraction, whereas no significant change was detected in the PAB240 fractions (Figure 6b). These results demonstrate that PEITC induced ‘WT-like' conformation to p53^R175^.

PEITC induces reactive oxygen species by disabling the GSH antioxidant system in cancer cells.^14, 22^ Redox changes affect the conformation of p53 WT.^23^ Therefore, we evaluated the effect of PEITC on GSH levels in SK-BR-3 cells. SK-BR-3 cells treated with PEITC (4 or 8 *μ*M) showed a decrease in the GSH levels as compared with the DMSO control (Figure 6c). Co-treatment of SK-BR-3 cells with PEITC and reducing agent (3 mM *N*-acetylcysteine (NAC) or 500 units PEG-catalase) alleviated the effects of PEITC on proliferation and apoptosis, whereas a catalase-specific inhibitor 3-amino-1,2,4-triazole (ATZ) enhanced it (Figures 6d–f). p53^R175^-knockdown SK-BR-3 cells did not display significant differences in apoptosis upon treatment with PEITC alone or PEITC in combination with ATZ or NAC (Figure 6f). Importantly, co-treatment with PEITC and oxidizing or reducing agents had no effect on p53^R175H^ degradation and aggregation (Supplementary Figure S3d), suggesting that redox changes are important for reactivation of p53^R175^ and inhibition of growth but not for restoring p53^R175^ conformation.

### Effects of PEITC on cell cycle and activation of ATM/CHK2

**Figure 7:**
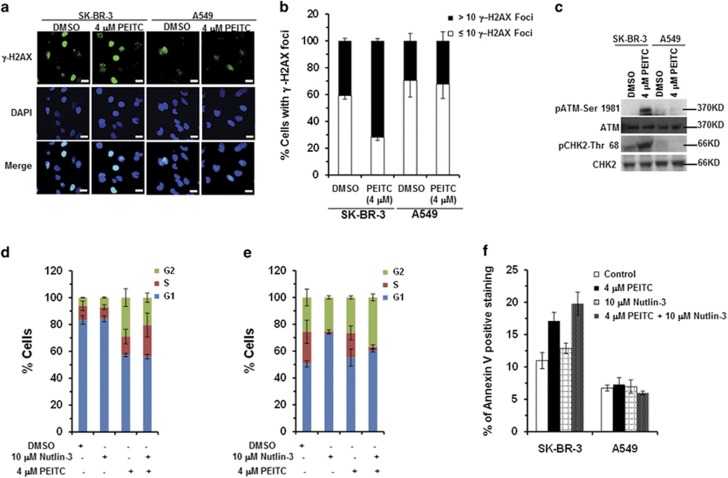
PEITC induces *γ*-H2AX foci, activates ATM/CHK2, G2/M- and S-phase arrest and apoptosis. SK-BR-3 and A549 cells treated with PEITC or DMSO for 3 days were stained with anti-*γ*-H2AX antibody. (**a**) Merged images show cells stained with anti-*γ*-H2AX antibody (green) and DAPI (blue). All scale bars represents a size of 20 *μ*m. (**b**) Percentage of cells with *γ*-H2AX foci (≤10 or >10, as indicated). (**c**) SK-BR-3 and A549 cells were treated with PEITC or DMSO for 4 h. Western blotting was performed using anti-pATM S1981 and anti-pCHK2 Thr68 antibodies. Blots were stripped and reprobed with anti-ATM and anti-CHK2 antibodies. (**d**) SK-BR-3 or (**e**) A549 cells were treated with PEITC, 10 *μ*M Nutlin-3 or both for 24 h and analyzed by flow cytometry. (**f**) SK-BR-3 and A549 cells were treated with 4μM PEITC, 10 *μ*M Nutlin-3 or both for 24 h. Apoptosis was measured by Annexin-V staining using a BD LSRFORTESSA instrument

WT p53 has a role in DNA repair and in maintaining genomic stability. As PEITC restored transactivation functions to p53^R175^ and exerted oxidative stress on SK-BR-3 cells, we evaluated its effect on DNA damage.^24^ SK-BR-3 cells treated with 4 *μ*M PEITC displayed an ∼1.8-fold increase in *γ*-H2AX foci as compared with the DMSO control, indicating the accumulation of DNA double-strand breaks (DSBs), whereas no differences were detected in A549 cells (Figures 7a and b). Further, p53^R175^-knockdown SK-BR-3 cells showed no difference in the number of *γ*-H2AX foci in cells treated with PEITC or DMSO (Supplementary Figures S4a and b), suggesting that accumulation of DSBs was p53^R175^ dependent.

DSB activates the ataxia telangiectasia mutated (ATM) serine/threonine protein kinase, which then phosphorylates downstream signaling targets. The p53^R175H^ inactivates the ATM-dependent DNA-damage response and induces genetic instability, whereas p53-null cells are efficient in ATM activation.^25^ Autophosphorylation of ATM at S1981 was detected in H1299 cells treated with PEITC (Supplementary Figure S4c). Therefore, we examined whether the reactivation of p53^R175^ to ‘WT-like' p53 abolishes its ability to inhibit the activation of the ATM/Checkpoint kinase 2 (CHK2) pathways. SK-BR-3 cells treated with 4 *μ*M PEITC showed pATM-S1981 and pCHK2/Thr68 compared with the DMSO control (Figure 7c), suggesting that the absence of inhibition of ATM/CHK2 by p53^R175H^ leads to the reactivation of the DNA-damage response. The absence of pATM-S1981 and pCHK2-Thr68 in A549 cells treated with PEITC was consistent with the *γ*-H2AX foci data (Figures 7a and b). Co-treatment of SK-BR-3 cells with PEITC and ATZ had no effect on pATM-S1981, whereas co-treatment with PEITC and NAC or PEG-Catalase completely abolished PEITC-induced ATM phosphorylation (Supplementary Figure S4d), suggesting that the reactivation of DNA-damage response by PEITC depends on redox state of the cells.

Next we examined whether reactivated p53^R175^ mutant affects cell cycle progression. SK-BR-3 cells treated with PEITC (4 *μ*M) displayed a significant increase in G2/M and S phases at 24 h (Figure 7d) and S-phase delay at 72 h (Supplementary Figure S5a), suggesting that PEITC inhibited cell proliferation by delaying cells not only in G2/M phase but also in the S phase. A549 cells treated with PEITC (4 *μ*M) showed an increase in the G1 phase at 24 h, and no change at 72 h (Figure 7e; Supplementary Figure S5b). No significant delay in S and G2/M phases was observed in p53^R175^-knockdown SK-BR-3 cells treated with PEITC (Supplementary Figure S5c), suggesting that the delay in cell cycle progression was p53^R175^ dependent.

We also examined the effects of Nutlin-3 and PEITC co-treatment on cell cycle progression and apoptosis. SK-BR-3 cells co-treated with 10 *μ*M Nutlin-3 and 4 *μ*M PEITC displayed remarkable increase in S-phase population (Figure 7d and Supplementary Figure S5a) and apoptosis (Figure 7f and Supplementary Figure S5d) at 24 and 72 h, respectively, compared with cells treated with PEITC or Nutlin-3 alone, indicating that Nutlin-3 exhibited a synergistic effect. Whereas, in A549 cells, Nutlin-3 treatment alone or in combination with PEITC resulted in a significant G1-phase delay at 24 h and G1 and G2/M-phase delays at 72 h (Figure 7e and Supplementary Figure S5b). Nutlin-3 alone induced apoptosis in A549 cells (Supplementary Figure S5d), demonstrating that the observed effects of Nutlin-3 were WT p53 specific. Together, these results demonstrate that restoration of the transactivation functions to p53^R175^ by PEITC and reactivation of the DNA-damage response culminates in apoptosis.

### PEITC reactivates p53^R175H^*in vivo* and inhibits SK-BR-3 xenograft tumor growth

**Figure 8:**
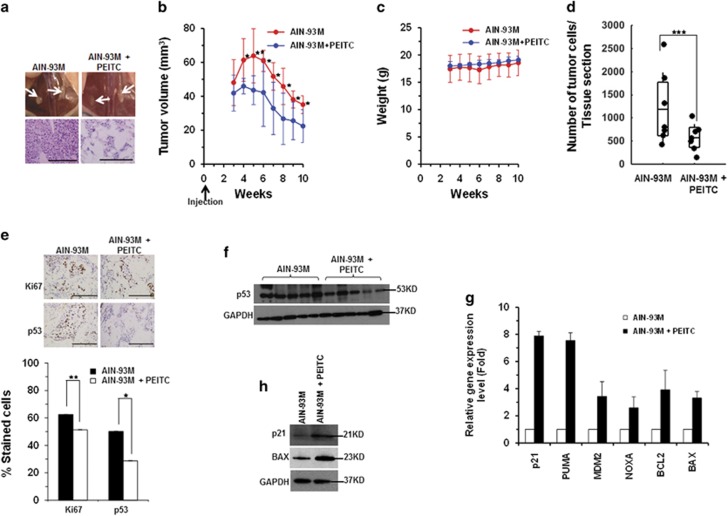
PEITC induces p53^R175H^ mutant reactivation *in vivo* and inhibits xenograft tumor growth. (**a**) Representative images of mouse mammary fat pads (upper panel), and H&E staining (lower panel). All scale bars represents a size of 200 *μ*m. (**b**) Tumors were measured with Vernier calipers, and tumor volumes were calculated. Formula *L* × *W*^2^ × 0.523 (**P≤0.009 and *P≤0.03; *n*=7). (**c**) Animal weights (g) were measured weekly. (**d**) Distribution of the animals based on the average number of tumor cells per tissue section in the control and PEITC groups (***P≤0.00026; *n*=7). (**e**) Representative images of xenograft tumor tissue stained for Ki67 (**P≤0.007) and p53 (*P≤0.033) (upper panel) and quantitation of positive cells (lower panel) (*n*=7). Results are expressed as ±S.D. All scale bars represents a size of 200 *μ*m. (**f**) Western blotting analysis of p53 expression levels in the xenograft tumors from the PEITC and control animal groups. Blot is representative of the 12 tumor tissue lysates analyzed from each group. (**g**) qRT-PCR (*n*=4) of p53-regulated genes in the PEITC and control animal groups. (**h**) Western blotting of p21 and Bax expression in SK-BR-3 xenograft tumors *in vivo*

The ability of PEITC to inhibit tumor growth in the SK-BR-3 xenograft mouse model was evaluated. A statistically significant inhibition of tumor growth (*P*<0.05) was observed in mice on the PEITC diet (5 *μ*mol/g AIN-93M)^26^ as compared with those on the control diet (Figures 8a and b and Supplementary Figure S6a). The decrease in tumor volumes in the control group after week 6 could be explained by the nonaggressive phenotype of the SK-BR-3 cells^27^ or by the immune response of the nu/nu mice as immune cells were detected in the hematoxylin and eosin (H&E)-stained sections (Supplementary Figure S6b). No difference in body weights was observed between the groups (Figure 8c). These results demonstrate that PEITC has antitumor activity in the SK-BR-3 xenograft model.

Histological examination revealed that tumor cells were remarkably depleted in the PEITC group (Figures 8a and d). A statistically significant reduction in Ki67- and p53-mutant-stained cells was detected in tumors from animals on the PEITC diet (Figure 8e). To assess whether PEITC induces p53^R175H^ mutant reactivation *in vivo*, we examined p53 protein in the tumor tissues. Because of the limited tumor tissues, especially from the PEITC-treated animals, the tumors were randomly divided for immunoblotting and quantitative real-time PCR (qRT-PCR) analysis. A significant reduction of p53 mutant protein levels was detected in animals on PEITC diet (Figure 8f). The variability in the reduced levels of p53^R175H^ in treated mice could be due to the inherent differences in this animal model. However, the p53 levels were consistently higher in the control group. Elevation in mRNA of p53-regulated genes and increase in p21 and Bax proteins were detected in animals fed PEITC diet (Figures 8g and h). These results provide evidence for the p53 mutant reactivation *in vivo* and the inhibition of SK-BR-3 xenograft tumor growth by PEITC.

## Discussion

Reactivation of the transactivation functions to p53 mutants presents a promising strategy to target cancer cells selectively. The reactivation of p53 has been shown in mouse model.^28^ Synthetic small molecules that restore the p53 point mutant to a transcriptionally competent form have been identified.^29, 30^ However, studies exploring the potential of dietary-related molecules targeting p53 mutants are scarce. PEITC selectively depletes p53 mutant protein and not the WT.^15^ In this study, we revealed a novel mechanism for PEITC that it inhibits cell proliferation and induce apoptosis by reactivating p53^R175^ to its WT function, resulting in the selective elimination of these cells. Similar effects were confirmed with human H1299 and MEF-derived p53^R175^-transfectant cells.

Previous studies have shown that in the WT p53 cells PEITC induced apoptosis in a p53-dependent manner.^8^ However, in p53-deficient cells PEITC is shown to induce apoptosis by activating extracellular signal-regulated kinases (ERK1/2).^12^ Thus the reduced cell viability and increased apoptosis in both p53^+/+^ and p53^−/−^, H1299 cells and MEF, by PEITC is consistent with the notion that pathway(s) independent of p53 mutational status also exists,^8, 12^ such as depletion of tubulin in both WT and p53 mutant cells.^31^

PEITC restored the transactivation functions to p53^R175^. Besides the antiapoptotic regulator BCL2, PEITC induced the expression of several pro-apoptotic targets, including the members of BH3-only class. Importantly, BCL2 failed to suppress the cell death induced by PEITC. p53 regulates BCL2 family members by engaging multiple transactivation-dependent and -independent effectors.^32^ It will be interesting to study the crosstalk between the different genes in PEITC-treated cells that ultimately result in the cell death.

ZnCl_2_ enhanced the antiproliferative activity of PEITC and also significantly increased the PAB1620 (WT-specific) fractions. Although, our results support that the reactivated p53^R175^ could bind zinc, thereby allowing its proper folding, further experiments are required to understand the mechanism of zinc binding and the enhanced efficiency of PEITC. A metallochaperone function has been demonstrated for a small molecule that could restore WT-like conformation to the p53^R175^.^30^ Further studies are required to discern the role of PEITC as a source of zinc for the reactivated p53^R175^. If PEITC indeed functions as a metallochaperone, it will be interesting to determine its effects on other zinc-binding mutants of p53, for example, C176F and C242F.

PEITC induced oxidative stress in SK-BR-3 cells, which may be exacerbated by increased ROS in p53 mutant cells (as p53-deficient cells are known for increased intracellular ROS^33^). Although induction of ROS had no effect on the restoration of p53^R175H^ conformation, the elevated oxidative stress was responsible for the activation of the restored p53^R175^ and induction of apoptosis. In support of this, ATZ enhances the antiproliferative ability of PEITC, whereas PEG-Catalase or NAC inhibits it.

As a proof-of-principle, we demonstrated that p53^R175H^ can be reactivated *in vivo* by dietary PEITC. We found 1.13±0.15 *μ*M ITC in the blood samples of the mice fed PEITC. Pharmacokinetic studies in humans have shown that after consuming ∼50 g of uncooked watercress (approximately equivalent to 40 mg PEITC) a peak concentration of 1 *μ*M PEITC can be reached in the plasma.^34, 35^

Reactivation of mutant p53 has been shown previously in xenograft models under ‘chemotherapeutic settings' where tumors are formed before the systemic administration of the drugs.^29, 30^ In this study, the inhibitory effect of PEITC was demonstrated under conditions that mimic cancer ‘chemopreventive settings'. In this bioassay, the animals were fed diets containing PEITC before the injection of the p53 mutant cells and formation of tumors, and mutant cells were then injected to mimic the presence of ‘cancerous' or ‘initiated' cells. Mice fed PEITC displayed a statistically significant decrease in tumor volumes, inhibition of proliferation and depletion of the p53^R175H^ *in vivo*. The elevated mRNA of p53 target genes from mice fed PEITC provides evidence for p53^R175H^ reactivation.

Significant efforts have been made to identify small molecules as therapeutics from chemical libraries aimed at reactivating p53 mutants. However, target-based prevention studies by dietary compounds are scarce. Our study elucidates a novel mechanism for PEITC, by preferential targeting of p53^R175^. Given that the R175 mutation is the third most common missense mutation among p53 mutants in human cancers, with an estimated 5.1% frequency of occurrence,^36^ PEITC may be developed as a lead compound for tumors with p53^R175^. Mutations in the p53 gene may occur at different phases during tumorigenesis, such as in the late stages of pancreatic neoplasia,^37^ hepatocellular carcinoma,^38^ prostate cancer^39^ and so on but in the early phases of ductal carcinoma *in situ* (DCIS), a precancerous lesion of breast^40^ and liver cancer.^41^ The occurrence of p53 mutations before the development of invasive breast cancer, particularly in DCIS, suggests the potential of PEITC in the prevention of breast cancer. Besides p53^R175^, PEITC inhibited the proliferation and induced apoptosis of cell lines with other hotspot mutants, including DNA contact mutants. More studies are needed to understand the mechanisms of growth inhibition of these mutants by PEITC. Nevertheless, the finding that a dietary-related compound restores the ‘WT-like' conformation and functions to the p53^R175^ opens up an opportunity for implementing a practical and effective target-based strategy for cancer prevention and treatment.

## Materials and Methods

### Cell lines

HOP92, OVCAR3 and SW620 were ordered from NCI DTP, DCDT Tumor Repository, Fredrick, MD, USA. H1299, HT29, A549, MDA-MB-231, AU565, SK-BR-3 and MCF7 were obtained from Tissue Culture Source Resource, Georgetown University, Washington, DC, USA. All the cell lines were negative for mycoplasma and cultured in RPMI 1640 with 10% FBS. Normal colon cells CCD841 purchased from ATCC (Manassas, VA, USA) were cultured in Eagle's minimal essential medium with 10% FBS. 3T3 Balb/c fibroblasts (p53^+/+^) were cultured in Dulbecco's modified Eagle's medium with 10% FBS. (10)3 (p53^−/−^) MEFs and (10)3-derived MEFs with p53 mutations ((10)3/175 and (10)3/273) were cultured in Dulbecco's modified Eagle's medium with 10% FBS and 400 *μ*g/ml G418. The MEF (10)3 and its derivative cells with human p53 mutations at residues R175 and R273 were previously derived^3^ and were a gift from Dr. Darren R Carpizo.

### Cell proliferation assays

The effect of PEITC on SK-BR-3 cell proliferation was determined by using the WST-1 assay (Roche, Indianapolis, IN, USA) as described previously.^42^ Briefly, PEITC was diluted in DMSO so that 10 *μ*l of diluted stock in a 1-ml aliquot of SK-BR-3 cells (40 000 cells/ml) yielded a desired concentration of PEITC at 1% DMSO. SK-BR-3 cell cultures containing PEITC were plated onto a 96-well microtiter plate at 4000 cells per well in duplicate. As a control, 4000 cells per well were seeded in medium containing 1% DMSO in duplicate. For background subtraction, wells lacking cells but containing medium were used. Plates were incubated at 37 °C for 3 days, followed by the addition of WST-1 reagent for 2 h. OD_450_ was measured using a microplate reader (Promega, Madison, WI, USA). Percentage of cell proliferation was calculated as the ratio of OD_450_ values obtained for respective cells grown in the presence of PEITC compared with the presence of DMSO. Similar assays were performed to determine the effect of PEITC on proliferation of H1299, HOP92, AU565, OVCAR3, SW620, HT29, A549, MCF7, CCD841 and SK-BR-3 cells transfected with p53 siRNA or NS siRNA, HOP92 cells transfected with p53 siRNA or NS siRNA, A549 cells transfected with p53 siRNA or NS siRNA, AU565 cells transfected with p53 siRNA or NS siRNA, SK-BR-3 cells transfected with ATG5 siRNA or NS siRNA, H1299 transfected with pcDNA3, H1299 transfected with pcDNA3-p53R175, H1299 transfected with pcDNA3-p53R273 and H1299 transfected with pcDNA3-wtp53, 3T3 Balb/c fibroblasts, (10)3 MEFs, (10)3/175 MEFs and (10)3/273 MEFs.

To determine the effect of the reducing agents, NAC or PEG-Catalase on PEITC activity, SK-BR-3 cells were treated with the indicated concentrations of PEITC, reducing agent or both. Cell proliferation was then measured by performing WST-1 assays as described previously. Similarly cells were either co-treated with PEITC and 2 mM ATZ or PEITC alone and cell proliferation was determined. To determine the effect of zinc on PEITC activity, SK-BR-3 cells were treated with the indicated concentrations of PEITC, zinc or both. Cell proliferation was then measured by performing WST-1 assays as described previously.

### Transfection in cells

The p53 siRNA was obtained from SMARTpool (Thermo Scientific/Dharmacon, Lafayette, CO, USA). The siRNA was transfected using Lipofectamine 2000 following the manufacturer's protocol (Invitrogen, Thermofisher Scientific, Pittsburgh, PA, USA). Briefly, cells were plated to 50–60% confluence in 10-cm dishes 24 h before transfection. The siRNA (0.430 nmol) was mixed with 43 *μ*l of Lipofectamine 2000 in 1 ml of Opti-MEM (Invitrogen). The mixture was added to cells that subsequently were incubated for 6 h. After 24 h, a second transfection was performed similarly. Seventy-two hours after the initial transfection, cells were harvested for preparing lysate or were treated with PEITC or DMSO at the indicated concentrations, and cell proliferation was measured using WST-1 reagent (Roche) as described previously for SK-BR-3 cells. For ATG5 knockdown in SK-BR-3 cells, ATG5 siRNA (Santa Cruz Biotechnology, Dallas, TX, USA) was transfected as described previously except that a single transfection was carried out for 6 h. After 24 h, transfected cells were harvested for preparing the lysates or were treated with PEITC for 4 h before lysates were prepared.

The plasmids pcDNA3, pcDNA3-wtp53, pcDNA3-p53R175 and pcDNA3-p53R273 were transfected using Lipofectamine 2000 following the manufacturer's protocol (Invitrogen). Briefly, cells were plated to 50–60% confluence in 10-cm dishes 24 h before transfection. The plasmid (14 *μ*g) was mixed with 43 *μ*l of Lipofectamine 2000 in 1 ml of Opti-MEM (Invitrogen). The mixture was added to cells that subsequently were incubated for 6 h. After 24 h, transfected cells were treated with PEITC for WST-1 assay or Annexin-V staining as described. The transfected cells were maintained in RPMI 1640 with 10% FBS and 400 *μ*g/ml G418.

### ELISA

*Escherichia coli* cells (BL21DE3) transformed with pGeX4T1-mutp53R175H vector were grown at 37 °C in LB medium containing 100 *μ*g/ml ampicillin to an optical density at 600 nm of 0.4. The expression of recombinant protein was induced by the addition of 0.5 mM isopropyl-1-thio-*β*-galactopyranoside for 3 h at the same temperature under vigorous shaking. Bacteria were pelleted and lysed in lysis buffer (250 mM Tris-HCl, pH 7.5, 1 mM EDTA, 150 mM NaCl, 1% Triton X-100, 0.5% Nonidet P-40, 0.1% Tween 20, 0.2% SDS, 1 M dithiothreitol (DTT) and protease inhibitors) by freeze–thaw cycles (three times) followed by probe sonication (three cycles of 1 min each). Sonicate was clarified by centrifugation at 18 500 × *g* for 30 min at 4 °C. The supernatant was transferred to a fresh tube and saved. The pellet was resuspended in sarcosyl buffer (lysis buffer+2% sarcosyl) followed by probe sonication (three cycles of 1 min each). The supernatant fractions from both the steps were diluted 1 : 1 in 1 × PBS and were incubated with glutathione-Sepharose beads (Sigma, St. Louis, MO, USA, G 4510) for 2 h at 4 °C with constant rotation. After several washes in PBS, protein was eluted in elution buffer (100 mM Tris-HCl, pH 8.0, 10 mM GSH (Sigma, G4251), 300 mM NaCl, 1 mM DTT and protease inhibitors). Twenty-five nanograms of recombinant GST-mutant p53^R175H^ (diluted in coating buffer 100 mM Tris-Cl, pH 7.5, 300 mM NaCl) was incubated with DMSO or 4 *μ*M PEITC for 1 h on ice. The protein samples were then applied to the ELISA plates and incubated at 4 °C for 2 h. The wells were washed with 1 × PBST (containing 0.05% Tween-20) and blocked by 5% skim milk at 4 °C for 2 h. Wells were rinsed and mouse primary antibody (PAB240 or PAB1620) diluted 1 : 1000 in 1 × PBST was added followed by incubation at 4 °C overnight. Wells were washed with 1 × PBST followed by addition of secondary anti-mouse HRP antibody at 4 °C for 1 h. Wells were rinsed with 1 × PBST and substrate was added (SuperSignal ELISA Pico Chemiluminescent Substrate, Thermo Scientific) followed by measuring chemiluminescence at 450 nM. As a control, purified GST only and purified WT p53 (Thermo Scientific) were used. To determine the effect of ZnCl_2_, 25 ng of recombinant GST-mutant p53^R175H^ (diluted in coating buffer 100 mM Tris-Cl, pH 7.5, 300 mM NaCl) was incubated with 2.5 *μ*M ZnCl_2_ or 4 *μ*M PEITC alone or in combination for 1 h on ice. ELISA assay was then performed as described.

### Annexin-V staining

The Annexin-V staining was carried out in accordance with the manufacturer's instructions (Biolegend, San Diego, CA, USA). In brief, SK-BR-3, A549, MDA-MB-231, OVCAR3, H1299 transfected with pcDNA3, H1299 transfected with pcDNA3- p53R175, H1299 transfected with pcDNA3-p53R273 and H1299 transfected with pcDNA3-wtp53, 3T3 Balb/c, (10)3 (10)3/175 and (10)3/273 cells were treated with PEITC as indicated or DMSO as a control for 3 days. Cells were harvested by scraping, washed once with 1 × PBS and resuspended in 0.5 ml Annexin-V-binding buffer. Cells were collected by centrifugation, 5 *μ*l of the fluorochrome conjugated Annexin-V was added in the residual buffer and cells were incubated at room temperature (RT) in the dark for 15 min followed by the addition of 0.5 ml of Annexin-V-binding buffer and 5 *μ*l of PI staining solution (0.1 *μ*g/ml). Cells were then analyzed by a BD LSRFORTESSA instrument (BD Biosciences, San Jose, CA, USA). To determine the effect of Nutlin-3 treatment on apoptotic induction, SK-BR-3 and A549 cells were treated with PEITC, Nutlin-3 or both or DMSO at the indicated concentrations for 24 or 72 h. Cells were then prepared for Annexin-V staining as described previously. To determine the effect of the reducing agents NAC or PEG-Catalase, on PEITC-induced apoptosis, SK-BR-3, NS siRNA-transfected SK-BR-3 or p53 siRNA-transfected SK-BR-3 cells were treated PEITC, reducing agent or oxidizing agent alone or PEITC in combination with reducing or oxidizing agent. Cells were then prepared for Annexin-V staining as described previously.

SK-BR-3 cells transfected with ATG5 siRNA or NS siRNA were treated with DMSO or PEITC at the indicated concentrations for 72 h. To assay apoptosis, the cytoplasmic histone-associated DNA fragments indicative of ongoing apoptosis were measured quantitatively using the cell death detection ELISA PLUS photometric enzyme assay (Roche).

### Immunofluorescent staining

SK-BR-3, A549, H1299 and (10)3/175 cells were treated with PEITC (4 or 6 *μ*M) or 1% DMSO as a control for 6 h in slide chambers with four wells (ThermoFisher Scientific). Cells then were washed twice with 1 × PBS and fixed with formaldehyde (3.7%) at RT for 15 min. Fixed cells were washed four times with 1 × PBS and treated with 0.5% Triton X-100 (Sigma) at RT for 5 min. Cells were washed four times with 1 × PBS containing 0.5% Tween-20 and blocked with 10% goat serum (Sigma) overnight at 4 °C. Cells were washed four times with 0.1% Tween-20 and incubated with mouse PAB240 (1:300, Calbiochem, San Diego, CA, USA) or mouse PAB1620 (1 : 300, Calbiochem) that recognizes specifically the mutant or p53 WT, respectively, overnight at 4 °C. After four washes with 0.1% Tween-20, cells were incubated with Alexa Fluor 488-conjugated goat anti-mouse IgG (1 : 400, Invitrogen) at RT for 2 h. Cells were washed four times with 0.1% Tween-20 and coated with Prolong Gold Anti-Fade reagent (Invitrogen) containing DAPI. Coverslips were placed on the chamber slides, and cells were cured at RT in the dark for 24 h. Immunofluorescence analyses were performed with a Zeiss LSM 510 META NLO inverted Axiovert 200 M laser scan microscope (Thornwood, NY, USA) with a Plan-Apochromat 63 × 1.4 numerical aperture oil immersion objective lens. Images were captured using the Photomultiplier Tube detectors and analyzed using the Image J software (NIH, available at http://rsb.info.nih.gov/ij/). Fluorescent staining intensity was quantified using the Metamorph software (Sunnyvale, CA, USA).

To determine the effect of PEITC on γ-H2AX foci formation in SK-BR-3 cells transfected with p53 siRNA or NS siRNA or A549 cell lines, cells were treated with 4 *μ*M PEITC or 1% DMSO as a control at 37 °C for 3 days. Cells were fixed with formaldehyde and processed for immunostaining to detect *γ*-H2AX foci as described above, except that mouse anti-γ-H2AX monoclonal antibody (1 : 300, Upstate, EMD Millipore, Billerica, MA, USA) was used as a primary antibody.

### Co-immunoprecipitation

SK-BR-3, A549 and HOP92 cells were treated with the indicated concentration of PEITC or 1% DMSO as a control for 6 h. For preparation of cell lysates, cells were harvested and washed once with 1 × PBS, cell pellets were suspended in lysis buffer (20 mM Tris-Cl (pH 8.0), 137 mM sodium chloride, 10% glycerol, 1% NP-40, 2 mM EDTA) and Protease inhibitors (Roche, Indianapolis, IN, USA) and the cells were incubated on ice for 30 min. The cell suspension was centrifuged at 18 500 × *g* for 10 min at 4 °C, and the supernatant was collected. The supernatants were diluted in lysis buffer, and 200 *μ*g of the lysate was gently tumbled at 4 °C for 1 h with protein G-agarose beads (Roche). The lysates obtained after preclearing were then gently tumbled at 4 °C for 2 h with mouse PAB240 antibody (2 *μ*g, Calbiochem). Protein G-agarose beads were then added to the suspensions and incubation was performed for 2 h at 4 °C. The beads were washed four times with lysis buffer supplemented with protease inhibitors, and the immunoprecipitates were eluted by boiling in Laemmli buffer and resolved on 4–12% SDS-PAGE. Immunoprecipitated p53 was detected by western blotting using FL393 (Santa Cruz Biotechnology) as a primary antibody. For the secondary antibody, peroxidase-labeled anti-mouse IgG (1 : 2000, GE Healthcare, Pittsburgh, PA, USA) was used. The blot was developed using the ECL Prime Western Blot Detection Kit according to the manufacturer's protocol (Amersham, GE Healthcare). As a control, the blot was stripped and then reprobed with anti-p53 (DO-1) antibody (1 : 1000, Santa Cruz Biotechnology) or anti-GAPDH antibody (1 : 2000, Novus Biologicals, Littleton, CO, USA). The density of the p53 bands in the PEITC treated samples relative to that of DMSO control was determined using the Gene Tools software (Cambridge, England, UK).

### Lysate preparation and western blotting analysis

Different lysis buffers were used to prepare soluble, insoluble and WCL fractions. For lysate (soluble fraction) preparation, cells were harvested and washed twice with 1 × PBS. RIPA buffer (10 mM sodium phosphate (pH 7.2), 300 mM NaCl, 0.1% SDS, 1% Nonidet P-40, 1% deoxycholate, 2 mM EDTA) was added to the cells, and the cells were incubated on ice for 30 min. Then the cell suspension was centrifuged at 18 500 × *g* for 10 min at 4 °C and the supernatant was collected, unless mentioned otherwise. The remaining pellet was defined as insoluble fractions. Insoluble fractions were dissolved in 2% SDS lysis buffer (65 mM Tris-HCl (pH 8.0), 150 mM NaCl, 2% SDS, 50 mM DTT). For WCL fractions, cells were harvested and the pellets were dissolved in 2% SDS lysis buffer as described previously. The fractions were collected by centrifuging the lysate at 18 500 × *g* for 10 min at 4 °C. Then 30–50 *μ*g of the lysates were loaded on 4–12% SDS/PAGE. Protein was transferred onto a PVDF membrane, and the blots were developed using the ECL Prime Western Blot Detection Kit according to the manufacturer's protocol (Amersham). The antibodies for p21, Bax, ATM, pATM S1981, CHK2, pCHK2 Thr68 and p53 (DO-1) were purchased from Santa Cruz Biotechnology and GAPDH antibody was from Novus Biologicals. The antibody for ATG5 (1 : 1000, Cell Signaling, EMD Millipore, Danvers, MA, USA) was a gift from Dr. Shivendra V Singh.

### Chromatin fractionation

SK-BR-3 cells were treated with the indicated concentrations of PEITC or DMSO as a control for 4 h. Cells were trypsinized and harvested by centrifugation at 500 × *g* for 5 min. Cell pellets were washed once with ice-cold PBS and transferred to 1.5-ml microcentrifuge tubes followed by centrifugation at 500 × *g* for 2 min. Pellets were stored at −80 °C prior to chromatin fractionation following the manufacturer's instruction (Subcellular Protein Fractionation Kit, Thermo Scientific) to generate nuclear soluble and chromatin-bound protein fractions. Ten micrograms of protein from the soluble nuclear extract and the chromatin-bound nuclear extract for the samples from DMSO- or PEITC-treated cells were resolved on 4–12% SDS-PAGE and transferred to PVDF membranes. Blots were probed with p53 (DO-1) antibody (1 : 1000, Santa Cruz Biotechnology). Histone H3 and TopoIIB, which served as markers for the chromatin and soluble nuclear fractions, respectively, were detected with rabbit anti-Histone H3 polyclonal (Thermo Scientific) and mouse anti-TopoIIB monoclonal (Santa Cruz Biotechnology) antibodies.

### RNA extraction and qRT-PCR

SK-BR-3, SK-BR-3 transfected with p53 siRNA or NS siRNA, H1299 and A549 cells were treated with 4 *μ*M PEITC or DMSO as a control for 4 h. RNA was extracted from the cells using a Qiagen RNeasy Kit (Qiagen, Valencia, CA, USA), cDNA was synthesized by using High Capacity RNA to cDNA Kit (Applied Biosystems, Invitrogen, Thermofisher Scientific) and the gene expression level was measured by qRT-PCR using TaqMan gene expression assays (Applied Biosystems, Invitrogen). The gene expression level is normalized with GAPDH, and the average is presented with S.D. from triplicates of repeated experiments. RNA was extracted from the SK-BR-3 xenograft tissues by using Qiagen RNeasy Kit and was processed further for qRT-PCR as described for the SK-BR-3 cells. The gene expression levels were normalized with GAPDH. Fold changes in the gene expression levels were calculated for each tumor from the treated group relative to the tumors from the control group and the average is presented with S.D.

### Measurement of glutathione level

The levels of reduced glutathione (GSH) and oxidized glutathione (GSSG) were measured using the GSH/GSSG-Glo Glutathione Assay Kit (Promega). Briefly, SK-BR-3 cells were treated with PEITC or DMSO as a control for 4 h. Cells were then processed for the glutathione assay as per the manufacturer's instructions (Promega).

### Luciferase reporter assay

WWP-Luc (p21/WAF1 promoter) plasmid encoding p53 WT-binding element in the p21 promoter region was a gift from Bert Vogelstein (Addgene plasmid no. 16451, Cambridge, MA, USA).^43^ It was transfected into SK-BR-3, HOP92, AU565, H1299 and MEF ((10)3/175 and (10)/273) cells, followed by treatment with PEITC (4 or 6 *μ*M, respectively) for 24 h. The cell lysate was made and the luciferase reporter assay was performed in accordance with the manufacturer's instructions (Luciferase assay, Promega).

### Cell cycle analysis

SK-BR-3, SK-BR-3 transfected with p53 siRNA or NS siRNA and A549 cells were treated with PEITC, Nutlin-3 or both or DMSO as a control for 24 or 72 h. Cells then were prepared for flow cytometric analysis. Briefly, cells were washed with PBS free of Ca^2+^ and Mg^2+^, trypsinized for 5 min and harvested by centrifugation at 190 × *g* for 3 min at 4 °C. Cells were washed once with PBS and the pellets resuspended in 1 ml of 70% ethanol and stored at –20 °C overnight. Cells were harvested by centrifugation at 420 × *g* for 10 min. The cell pellets were washed once with 1 ml cold PBS and resuspended in 1 ml freshly prepared PI staining solution (PBS with 0.1% Triton X-100, 0.05 *μ*g/ml propidium iodide, 0.1 mg/ml RNase (Sigma)). The cell suspension was incubated at RT for 30 min in the dark followed by incubation for 30 min at 4 °C. The samples were run on a Becton Dickinson FACS sorter (BD Biosciences, San Jose, CA, USA) and the data were analyzed using Mod Fit program (Verity Software House, Topsham, ME, USA).

### Detection of ATM and CHK2 phosphorylation upon PEITC treatment

Cells were treated with either DMSO, ATZ, NAC, PEG-Catalase or PEITC alone or PEITC in combination with ATZ or NAC or PEG-Catalase for 4 h. Cells then were harvested by centrifugation at 1600 × *g* for 10 min at 4 °C, washed once with PBS and resuspended in RIPA buffer (10 mM sodium phosphate (pH 7.2), 300 mM NaCl, 0.1% SDS, 1% Nonidet P-40, 1% deoxycholate and 2 mM EDTA) containing a protease and phosphatase inhibitor mixture and were incubated on ice for 30 min and centrifuged at 18 500 × *g* for 10 min at 4 °C. The supernatant was collected, and 200 *μ*g of the lysate was loaded on 4–12% SDS/PAGE. Following electrophoresis, protein was transferred onto a PVDF membrane, and blot was probed with anti-pATM Ser1981 antibody (1 : 500) (Santa Cruz Biotechnology) or anti-pCHK2 Thr68 antibody (1 : 500) (Santa Cruz Biotechnology). For the secondary antibody, peroxidase-labeled anti-mouse IgG (1 : 1000, GE Healthcare) was used. The blot was developed using the ECL Prime Western Blot Detection Kit following the manufacturer's protocol (Amersham). As a control, the blot was stripped and then reprobed with anti-ATM anti-body (1 : 500, Santa Cruz biotechnology) or CHK2 antibody (1 : 500, Santa Cruz biotechnology).

### Mouse SK-BR-3 xenograft model

Twenty female athymic nu/nu Balb/c mice (CAnN.Cg-Foxn1nu/Crl, 4–6-week old) were purchased from Charles River Laboratories (Wilmington, MA, USA). All *in vivo* studies and tumor harvest were performed in accordance with the Institutional Animal Care and Use Committee procedures and guidelines. Mice were weighed and housed under quarantine in polycarbonate cages (five mice/cage, equal average weight and variance among animals/cage) for 1 week. Mice were fed an AIN-93M diet and water *ad libitum* for 1 week of quarantine. After 1 week in quarantine, the mice were placed on either a control AIN-93M diet or an AIN-93M diet supplemented with PEITC (5 *μ*mol PEITC/g diet) (10 mice/group). Both diets were prepared by Research Diets (New Brunswick, NJ, USA). The number of animals was decided based on previous studies, which used the SK-BR-3 xenograft mouse model to generate statistically significant data, and the concentration of the PEITC in diet was chosen based on our previous bioassay in mice.^27, 44^ The PEITC concentration in the diet was confirmed to be 4.97±0.16 *μ*mol/g by ethyl acetate extraction followed by cyclocondesation reaction of 1,2-benzenedithiol with the ITCs as described.^34^ By single-factor ANOVA calculation, there was no statistically significant difference (alpha=0.05) between the PEITC samples. The food was replenished every alternate day. After 1 week of control and PEITC diets, the mice were injected with exponentially grown 2 × 10^6^ SK-BR-3 cells (suspended in 50 *μ*l of Matrigel) in their left and right mammary fat pads (‘cancer chemoprevention' settings). No mortality or death was observed over the course of the experiment. Tumor formation was assessed externally weekly, and tumor dimensions were measured with Vernier calipers for a 10-week bioassay period. Tumor volumes were calculated with the formula *L* × *W*^2^ × 0.523. The tumors were small in size and were not easily measurable externally. Because of the small size, some of the tumors showed variations in the tumor volume measurements over the weeks. For this reason, only the tumors (*n*=7) that followed a growth pattern without fluctuations were considered for the final tumor volume calculations. However, no outliers were detected by using the GraphPad software (GraphPad Software, Inc., La Jolla, CA, USA). All the animals were killed, and the tumors were removed and were confirmed by H&E staining as described below. Animal weights (g) were measured weekly. The ITC concentration in the blood of the animals collected at the time of necropsy was evaluated by cyclocondensation reaction of 1,2-benzenedithiol with ITCs as described.^34^ PEITC concentration was found to be 1.13±0.15 *μ*M (*n*=3) and 0.37±0.03 *μ*M (*n*=2) in the blood of the PEITC-treated and control animals, respectively. Blinding to the groups was not possible because of the different diets; experiments were, however, blinded to tumor harvest and histopathological analysis. H&E-stained sections were blindly examined by pathologist for the incidence of tumors.

### Histopathological analysis, immunohistochemistry, western blotting and qRT-PCR analysis of the SK-BR-3 tumor xenografts

H&E-stained slides were examined and tissues were confirmed as tumors by histopathological examination. Immunohistochemistry was performed at Histopathology and Tissue Shared Resources, Georgetown University following standard protocols. Briefly, tissues were sectioned at 5 *μ*m, de-paraffinized with xylenes and rehydrated through a graded alcohol series. Heat-induced epitope retrieval was performed by immersing the tissue sections at 98 °C for 20 min in 10 mM citrate buffer (pH 6.0) with 0.05% Tween. Immunohistochemical staining was performed using a horseradish peroxidase-labeled polymer from Dako (Carpinteria, CA, USA) (K4001, K4003) according to the manufacturer's instructions. Briefly, slides were treated with 3% hydrogen peroxide and 10% normal goat serum for 10 min each and exposed to primary antibodies p53 (DO-7) (1 : 800, Dako) for 1 h at RT and Ki-67 (1 : 15, Novus Biologicals) overnight at 4 °C. Slides were exposed to the appropriate HRP-labeled polymer for 30 min and DAB chromagen (Dako) for 5 min. Slides were counterstained with Hematoxylin (Fisher Scientific, Suwanee, GA, USA, Harris Modified Hematoxylin), blued in 1% ammonium hydroxide, dehydrated and mounted with Acrymount (StatLab, Baltimore, MD, USA). Consecutive sections with the primary antibody omitted were used as negative controls. The sections were examined under an Olympus BX61 microscope at × 200 magnification. Representative images were captured from the entire tumor section using a DP70 camera and DP70 software (Olympus, Waltham, MA, USA), and images were analyzed using Image J software (NIH). Further, because of the small size of the tumors, four sections per tumors were analyzed for determining the cell number. The sections were stained with different antibodies as mentioned and for each antibody 20 pictures were taken from different areas on slide to count the total cell number. The data presented for each tumor are the average of the total number of cells from different antibody stainings.

As we were limited by the amount of tumor tissue, especially from the mice on the PEITC-supplemented diet, we divided the tumors randomly to perform western blotting and qRT-PCR analyses. For western blotting analysis, the SK-BR-3 tumor xenograft (*n*=12) lysate from each group was prepared by homogenizing the tissue in 20w/v of lysis buffer (Pierce, Rockford, IL, USA). Twenty-five micrograms of the lysates were loaded on 4–12% SDS/PAGE, transferred onto a PVDF membrane and the blot was probed with p53 (DO-1) antibody as described previously. To perform qRT-PCR assay with the SK-BR-3 tumor xenograft tissues, RNA was extracted from tumor tissues (*n*=4) using the Qiagen RNeasy Kit and gene expression level was measured as described previously.

### Statistical analysis

Statistical differences in tumor volume and biological end points were evaluated with a two-tailed Student's *t*-test. Differences were considered statistically significant at *P*-values of ≤0.05. All statistical tests were two-sided.

## Abbreviations

PEITC: phenethyl isothiocyanate
WT: wild type
MEF: mouse embryonic fibroblast
ELISA: enzyme-linked immunosorbent assay
WCL: whole-cell lysate
ATG5: autophagy protein 5
CHQ: chloroquine
ZnCl_2_: zinc chloride
NAC: *N*-acetylcysteine
ATZ: 3-amino-1,2,4-triazole
ATM: ataxia telangiectasia mutated
CHK2: checkpoint kinase 2
qRT-PCR: quantitative real-time PCR
DCIS: ductal carcinoma *in situ*
