# Supplementary material for: Reactivation of mutant p53 by a dietary-related compound phenethyl isothiocyanate inhibits tumor growth
Source: Cell Death Differ. 2016 Jun 3;23(10):1615–27. doi: 10.1038/cdd.2016.48 (PMC5041190; doi:10.1038/cdd.2016.48)
Supplement: Supplementary Figure Legends [file cdd201648x1.doc]

**Supplementary Figure Legends**

**Supplementary Figure S1. Effects of PEITC on cell proliferation and apoptosis.** (**a**) A549, SK-BR-3, AU565 and HOP92 cell lines were transfected with p53 siRNA or NS siRNA for the indicated number of days as described in Materials and Methods. Effect of p53 siRNA on p53 expression levels was then determined by western blot analysis. Thirty μg of the cell lysate was resolved by SDS-PAGE and probed with p53 DO-1 antibody. Blots were stripped and reprobed with anti-GAPDH antibody. (**b**) HOP92 (**c**) AU565 cell lines transfected with p53 siRNA or NS siRNA were treated with DMSO (control) or the indicated concentration of PEITC for 3 d. Percent cell proliferation determined by the WST-1 assay. (**d**) Effect of PEITC on apoptosis. MDA-MB-231 and OVCAR3 cells were treated with DMSO or 4μM PEITC for 3 d. Apoptosis was measured by Annexin-V staining using a BD LSRFORTESSA instrument. (**e**) MEF’s cells (10)3 (p53^-/-^), (10)3/175, (10)3/273 and Balb/c 3T3 (p53^+/+^) were treated with DMSO or PEITC for 3 d. Percent cell proliferation was determined by the WST-1assay. (**f**) Effect of PEITC on apoptosis. (10)3, (10)3/175, (10)3/273 and Balb/c 3T3 (p53^+/+^) were treated with DMSO or 6μM PEITC for 3 d. Apoptosis was measured by Annexin-V staining using a BD LSRFORTESSA instrument.

**Supplementary Figure S2. Effects of PEITC on conformation of p53^R175^ mutant protein.** (**a**) (10)3/175 MEF cells were treated with DMSO (control) or 6μM PEITC for 6 h. Cell were then stained with conformation specific antibodies PAB240 (mutant-specific) and PAB1620 (WT-specific) as described in Materials and Methods. All scale bars represents a size of 20 μm (**b**) Immunoprecipitation of the p53 mutant protein from HOP92 cell lysates using PAB240 antibody. HOP92 cells were treated with 2μM PEITC for 4 h and cell lysates were prepared as described in Materials and Methods. Two hundred μg of the HOP92 cell lysate was incubated with PAB240 and immunoprecipitated using protein-G agarose beads. The immunoprecipitated proteins were resolved by SDS-PAGE and probed with FL393 antibody. Blots were stripped and reprobed with anti-GAPDH antibody.

**Supplementary Figure S3.** **Effects of PEITC on stability of p53 protein.** (**a**) Human tumor cell lines with hotspot p53 mutations were treated with DMSO (control) or the indicated concentration of PEITC for 4 h. Thirty μg of the cell lysate was resolved by SDS-PAGE and probed with p53 DO-1 antibody. Blots were stripped and reprobed with anti-GAPDH antibody. (**b**) SK-BR-3, HT29 and OVCAR3 cells were treated with 4 μM PEITC over a 24 h time course. Protein levels were determined by western blotting using p53 DO-1 and GAPDH antibodies. (**c**) Quantitation of the blots shown in (**b**). ** p ≤ 0.009 and * p ≤0.02, respectively. (**d**) SK-BR-3 cells were treated with DMSO, 8 μM PEITC, 2 mM ATZ, 3 mM NAC or 500 units PEG-Catalase alone or PEITC in combination with ATZ or NAC or PEG-Catalase for 4 h. Cells were harvested and soluble and insoluble fractions were prepared. Thirty μg of the soluble and insoluble lysate fractions were resolved by SDS-PAGE and probed with p53 DO-1 antibody.

**Supplementary Figure S4. PEITC induces γ-H2AX foci and activates ATM.** SK-BR-3 cells transfected with NS siRNA or p53 siRNA were treated with PEITC or DMSO for 3 d and were then stained with anti-γ-H2AX antibody. (**a**) Merged images shows cells stained with anti-γ-H2AX antibody (green) and DAPI (blue). All scale bars represents a size of 20 μm. (**b**) Percentage of cells with γ-H2AX foci (≤ 10 or > 10, as indicated). (**c**) H1299 cells were treated with DMSO or PEITC for 4 h. Western blotting was performed using anti-pATM-S1981 antibody. Blot was stripped and reprobed with anti-ATM antibody. (**d**) SK-BR-3 cells were treated with 2 mM ATZ, 3 mM NAC or 500 units PEG-Catalase alone in combination with 4 μM PEITC for 4 h. Western blotting was performed using anti-pATM-S1981 antibody. Blot was stripped and reprobed with anti-ATM antibody.

**Supplementary Figure S5. Effects of PEITC on cell cycle progression and apoptotic induction in SK-BR-3 cells.** SK-BR-3 (**a**) or A549 (**b**) cells were treated with 4μM PEITC, 10 μM Nutlin-3 or both for 24 h. Cells then were analyzed by flow cytometry. (**c**) SK-BR-3 cells transfected with NS siRNA or p53 siRNA were treated with DMSO or 4μM PEITC for 24 h. Cells then were analyzed by flow cytometry. (**d**) SK-BR-3 and A549 cells were treated with 4μM PEITC, 10 μM Nutlin-3 or both for 24 h. Apoptosis was measured by Annexin-V staining using BD LSRFORTESSA instrument.

**Supplementary Figure S6. Immune cells in SK-BR-3 xenograft tumors.** (**a**) Representative images of mouse mammary fat pads (Upper Panel) and H&E staining (Lower Panel). (**b**) Representative images of the H&E stained tumor tissue sections of PEITC or control diet fed animals showing immune cells (black circles) surrounding the SK-BR-3 xenograft tumors (T). All scale bars represents a size of 200 μm.
