# Supplementary figures and images for: Reactivation of mutant p53 by a dietary-related compound phenethyl isothiocyanate inhibits tumor growth
Source: Cell Death Differ. 2016 Jun 3;23(10):1615–27. doi: 10.1038/cdd.2016.48 (PMC5041190; doi:10.1038/cdd.2016.48)

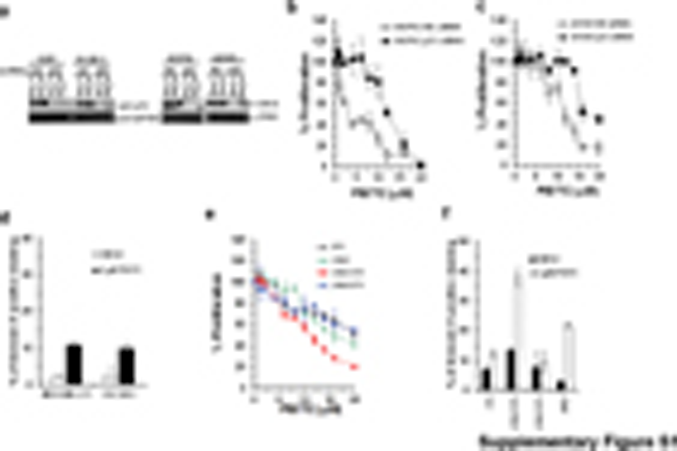

Supplement: Supplementary Figure 1 [file cdd201648x2.tif]

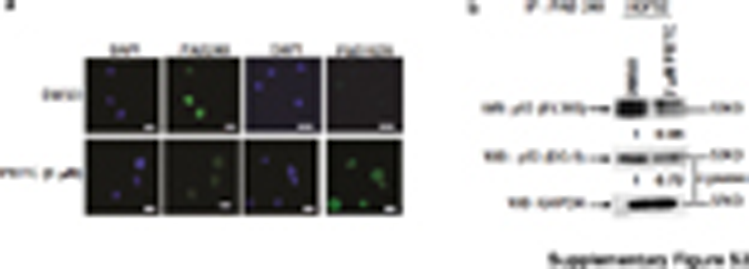

Supplement: Supplementary Figure 2 [file cdd201648x3.tif]

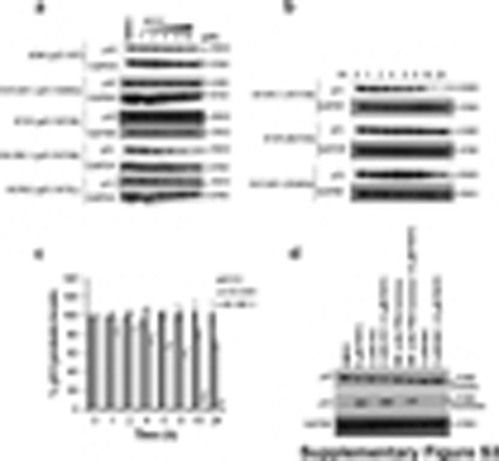

Supplement: Supplementary Figure 3 [file cdd201648x4.tif]

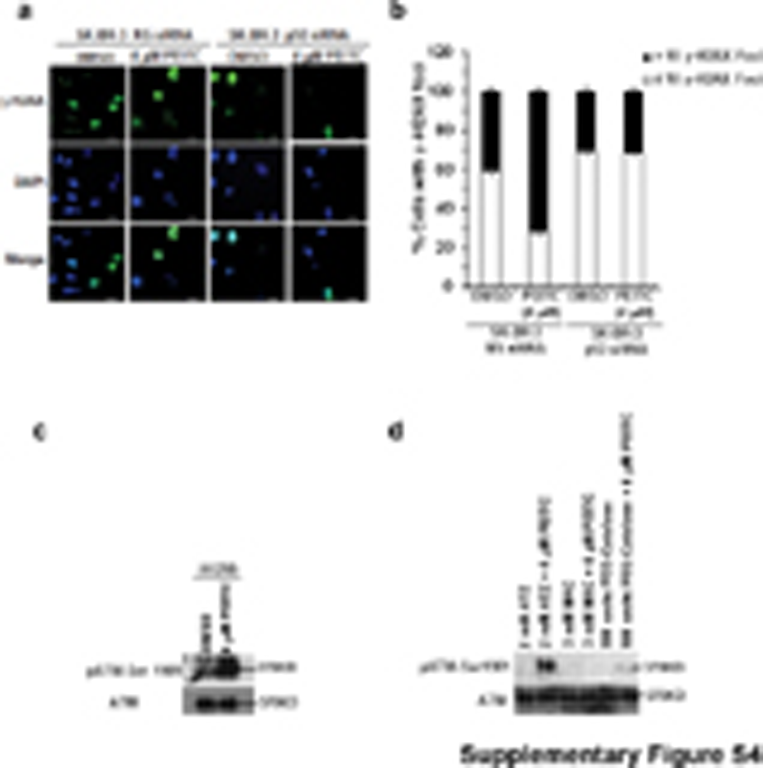

Supplement: Supplementary Figure 4 [file cdd201648x5.tif]

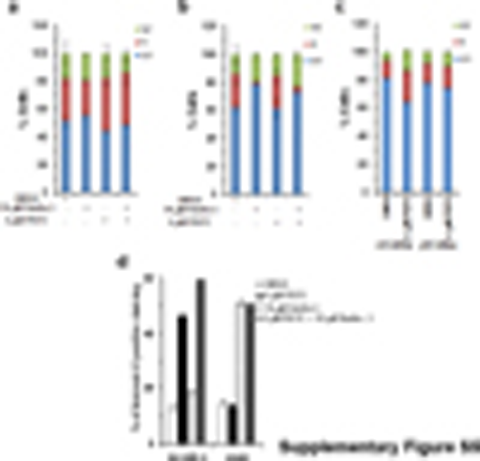

Supplement: Supplementary Figure 5 [file cdd201648x6.tif]

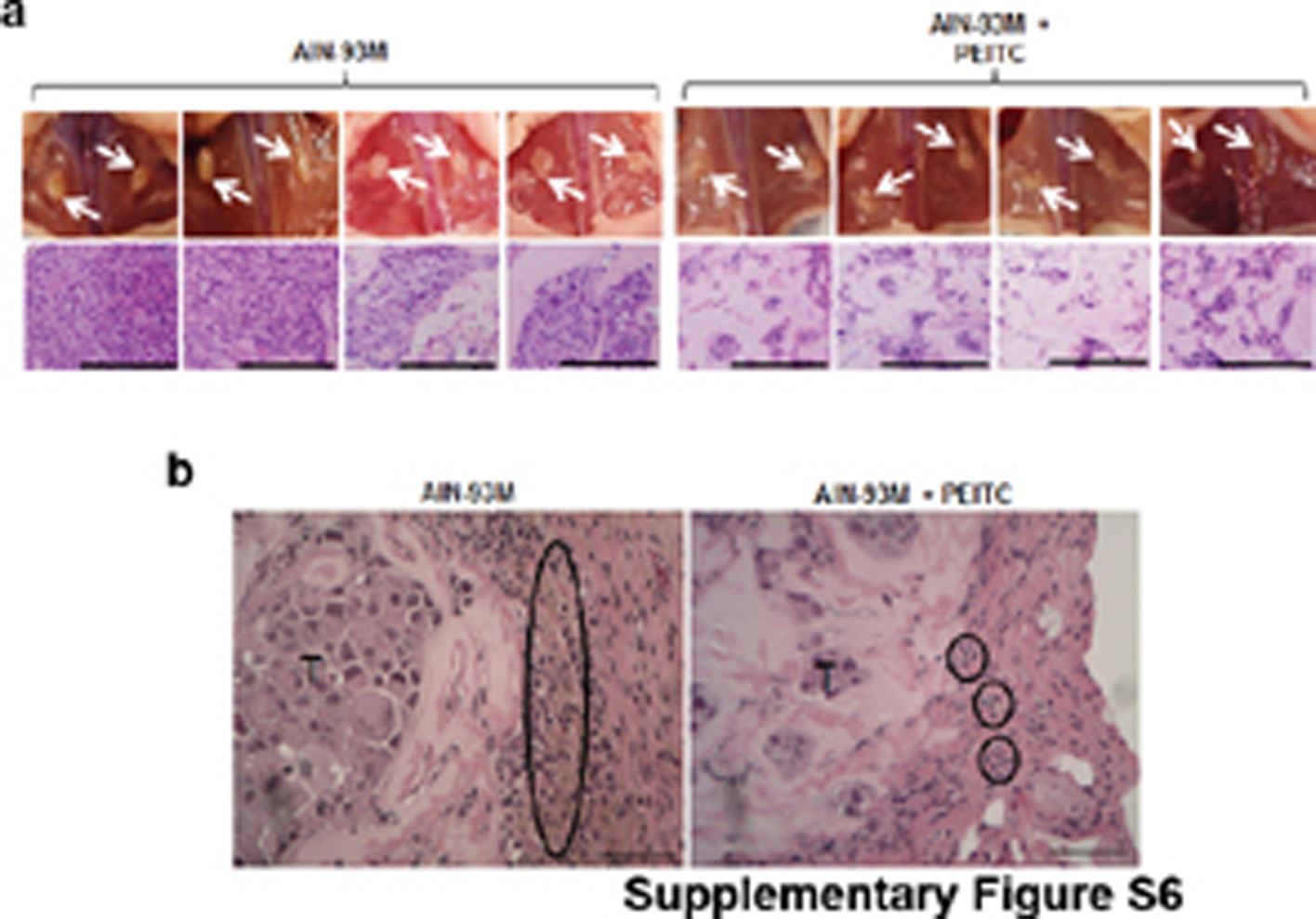

Supplement: Supplementary Figure 6 [file cdd201648x7.tif]
